# Impact of decreased insulin resistance by ezetimibe on postprandial lipid profiles and endothelial functions in obese, non-diabetic-metabolic syndrome patients with coronary artery disease

**DOI:** 10.1007/s00380-018-1319-x

**Published:** 2018-12-05

**Authors:** Akihiro Nakamura, Kenjiro Sato, Masanori Kanazawa, Masateru Kondo, Hideaki Endo, Tohru Takahashi, Eiji Nozaki

**Affiliations:** Department of Cardiology, Iwate Prefectural Central Hospital, 1-4-1 Ueda, Morioka, 020-0066 Japan

**Keywords:** Ezetimibe, Postprandial hyperlipidemia, Postprandial hyperinsulinemia, Insulin resistance, Flow-mediated vasodilation

## Abstract

The association between insulin resistance and lipid dysmetabolism after consuming a meal is unclear. We aimed at assessing the effects of ezetimibe on postprandial hyperlipidemia and hyperinsulinemia and to find out whether the medication improves endothelial function in obese metabolic syndrome (MetS) patients with coronary artery disease (CAD). We obtained oral fat loading test results (4 and 6 h after load) and brachial flow-mediated vasodilation (FMD) measurements before and 24 weeks after ezetimibe treatment initiation from 27 MetS patients with CAD and from 68 control patients with CAD alone. Serum triglyceride (TG) and insulin levels (2 h after the loading dose) were significantly higher in MetS patients than in control patients. The incremental areas under the curve (*i*AUCs) for these levels decreased significantly after ezetimibe treatment in MetS patients but not in control patients. Treatment with ezetimibe resulted in significant FMD changes in MetS patients (from 3.4 to 4.9%, *P* = 0.002), but not in control patients (from 5.1 to 5.4%, *P* = 0.216). When MetS patients were divided into two groups based on the median insulin *i*AUC reduction rate (higher group ≥ 34%, *n* = 14; lower group < 34%, *n* = 13), those in the higher group showed a significantly higher rate of change in the *i*AUCs of TG and FMD than those in the lower group (TG, 31.0% vs. 10.8%; *P* = 0.033; FMD, 39.2% vs. 9.8%; *P* = 0.037). These results suggest that ezetimibe may reverse insulin resistance, reducing lipid dysmetabolism after a meal and endothelial dysfunction in MetS patients with CAD.

## Introduction

Postprandial hyperlipidemia is characterized by pronounced and prolonged high serum levels of triglyceride (TG) and excess TG-rich lipoproteins with their partially hydrolyzed products (chylomicron and very low-density-lipoprotein remnants) [1–3]. This dysmetabolism plays a role in the progression or vulnerability of atheromatous plaques [1, 4, 5] and is a potent risk factor for coronary artery disease (CAD) independent of the low-density-lipoprotein cholesterol (LDL-C) levels [6, 7]. High serum insulin levels after a meal may also predispose to atherosclerosis development [8]. Both postprandial hyperlipidemia and hyperinsulinemia have been observed accompanying metabolic syndrome (MetS) [9], a pathological clustering of metabolic components including glucose intolerance and dyslipidemia [10] that increases the risk of cardiovascular diseases [11, 12].

Ezetimibe, a lipid-lowering drug that selectively inhibits intestinal cholesterol absorption by binding to the Niemann−Pick C1-like 1 (NPC1L1) protein [13], reduces the serum levels of LDL-C and fasting TG, especially when used with statins [14]. But reports indicating a postprandial hyperlipidemia reduction by ezetimibe [9, 15, 16] did not evaluate its effect on postprandial lipid dysmetabolism in patients with MetS. Insulin resistance (impaired insulin sensitivity) is an underlying mechanism of MetS [17, 18]. Ezetimibe also reduces insulin resistance marker levels like the homeostasis model assessment of insulin resistance (HOMA-IR) [19, 20]; however, little is known regarding its effects on postprandial hyperinsulinemia.

We previously reported that the insulin resistance showed a close relationship with postprandial hyperlipidemia in CAD patients with diabetes mellitus (DM) [21]. To examine whether insulin resistance could impact on postprandial hyperlipidemia or hyperinsulinemia even without DM, we enrolled MetS patients without DM (study group) and compared to non-MetS patients without DM who were served as control in this study. Our primary endpoint was to estimate the postprandial hyperlipidemia and hyperinsulinemia in study group, and the effect of ezetimibe on these conditions after the meal. Secondary endpoint was to examine whether flow-mediated vasodilation (FMDs) for assessment of endothelial function [22] could be improved in study group, and to determine the association between reduced postprandial hyperinsulinemia and postprandial hyperlipidemia or improved FMD.

## Methods

We conducted 24 week, prospective, open-label, single-center studies from June 2016 to September 2017 to examine the effects of ezetimibe on postprandial hyperlipidemia and its association with insulin resistance in men with MetS and CAD receiving statin therapy. We followed the principles of the Declaration of Helsinki, explained the protocol to the participants, and obtained signed written informed consents. The ethics committee of the Iwate Prefectural Central Hospital approved the study's protocol.

## Study patients

We enrolled 95 men who presented consecutively with stable angina pectoris on atorvastatin prescriptions (10 mg, daily once) and who had angiographically confirmed CADs. This group included 27 patients with MetS (MetS group, mean age 66.2 ± 9.8 years) and 68 patients without MetS matched by age (control group, mean age 67.5 ± 9.4 years) who were tested with loading doses of high-fat and high-glucose meals before and 24 weeks after ezetimibe treatment initiation. In this study, all patients were male to rule out any estrogen effects on postprandial lipid metabolism. We also excluded patients with: (1) type 1 or 2 DM; (2) gastrointestinal disease limiting drug absorption or partial ileal bypass; (3) major surgery within 6 months of enrollment, concomitant inflammatory disease, or malignant tumors; (4) congestive heart failure, active liver disease, or hepatic dysfunction (alanine aminotransferase or aspartate aminotransferase levels above the normal ranges); (5) concurrent therapy with long-term immunosuppressants; (6) familial hypercholesterolemia; and (7) those taking lipid-lowering medications without statins (e.g., eicosapentaenoic acid or docosahexaenoic acid therapy).

## Definitions

We defined MetS as the presence of two or more metabolic abnormalities of the following three components, in addition to visceral obesity (abdominal circumference ≥ 85 cm in males) based on the Japanese Committee for the Diagnostic Criteria of MetS [23]:

1. TG ≥ 150 mg/dL, high-density lipoprotein cholesterol (HDL-C) ˂ 40 mg/dL, and/or the use of medication for dyslipidemia.
2. Blood pressure ≥ 130/85 mmHg and/or the use of antihypertensive medication.
3. Fasting plasma glucose (FPG) ≥ 110 mg/dL and/or the use of medication for DM.

We excluded patients with DM because DM alone may be a risk factor for impaired postprandial lipid metabolism and shows a close association with postprandial hyperinsulinemia [21]. DM was diagnosed according to the American Diabetes Association (ADA) criteria [FPG level ≥ 126 mg/dL, a glycated hemoglobin A1c (HbA1c) level ≥ 6.5%, and/or the present use of hypoglycemic agents] [24]. DM and MetS were not present in control patients.

Patients with stable angina pectoris had a history of myocardial infarction, coronary artery bypass, percutaneous coronary intervention with or without stenting, or previous angiographically proven stenotic lesions ≥ 75% in a major epicardial coronary artery. They were also diagnosed as being in a stable condition when chest pain was brought on by exertion, resolved under nitrate therapy, and had no characteristic changes (frequency, severity, duration, time of appearance, and precipitating factors) for the previous 60 days [25].

## High-fat loading test and blood sampling

Patients were administered an oral high-fat and high-glucose meal [1003 kcal, 28.6 g of protein (11.4%), 62.4 g of lipid (56.0%), 80.7 g of carbohydrate (32.2%), and 320.5 mg of cholesterol (0.4%)] [21] for breakfast before and 24 weeks after ezetimibe treatment initiation. The patients were instructed were prohibited from exercising or consuming food, caffeine, vitamins, or alcohol within 12 h before the loading test starts. Patients were requested to consume the meal within 30 min under stable conditions. Blood samples were collected during the fasting state just before the test and at 0, 2, 4, and 6 h after loading. Sera were immediately separated by low-speed centrifugation (3000 rpm for 15 min at 4 °C) and kept at − 80 °C until processed. A commercial laboratory (SRL, Tokyo, Japan) determined serum TG levels by enzymatic methods, serum LDL-C and HDL-C levels by a direct method, serum apolipoprotein A-I and apolipoprotein B (Apo B) levels by an immunoturbidity method, and serum remnant-like particle cholesterol (RLP-C) levels by the immunoaffinity isolation method. We avoided the Friedewald formula calculation for serum LDL-C levels because the postprandial TG levels were predicted to be 400 mg/dL.

Plasma glucose and insulin levels were also measured before and after the oral fat meal ingestion. Plasma insulin levels were determined using chemiluminescent enzyme immunoassay and HbA1_C_ levels using high-performance liquid chromatography at our hospital laboratory. Each fasting value was obtained before the loading test. The lipid and glucose parameter areas under the curve (AUCs) were also calculated using the trapezoidal method, and incremental AUCs (*i*AUCs) were calculated as total AUC minus the area under the basal value. We estimated the following insulin resistance parameters: (1) HOMA-IR (2) fasting insulin level (3) insulin level 2 h after the test, and (4) *i*AUC_0−6 h_ for plasma insulin [26]. HOMA-IR was calculated as [FPG (mg/dL) × fasting plasma insulin (µIU/mL)/405] [27].

## Brachial artery FMD measurement

We measured FMD for assessing endothelium-dependent vascular function in the brachial artery after blood sampling 4 h after the oral fat loading test. All patients sat in a quiet, air-conditioned room with a stable temperature of 23 °C ± 1 °C for 30 min before and during the measurement. Two trained ultrasonographers blinded to the study details measured brachial FMDs with a semi-automated edge detection system device (UNEXEF18G; UNEX, Nagoya, Japan) in accordance with guidelines [28]. Briefly, the technician scanned the right brachial artery with a 10 MHz linear array transducer for longitudinal and transverse high-resolution images with the rested patient in a supine position. A sphygmomanometric cuff attached to the UNEXEF18G was positioned around the right forearm, and the ultrasonographer obtained artery images on the proximal portion of the antecubital fossa. After obtaining baseline images, the cuff was inflated to at least 50 mmHg above the systolic blood pressure for 5 min and then deflated. The ultrasonographer then obtained post-deflation arterial images similar to those for reactive hyperemia and measured artery diameters for 2 min with R-wave synchronized automated edge-detection software. Brachial artery FMD was calculated as ([maximum − baseline diameter]/baseline diameter) × 100%. We assessed inter- and intra-reader variabilities in 80 randomly selected and blinded FMD scan images. We found no statistically significant differences between the first and second %FMD measurements in both observers (− 0.15%; 95% CI −1.09 to 0.72%; 0.06%, 95% CI −0.84 to 0.51%) or between the observers (− 0.07%, 95% CI −0.71 to 0.57%). The repeatability of the measurement between the two observers was high (*r* = 0.86).

### Statistical analysis

We determined sample size based on the published estimated FMDs [29] and assumed a mean %FMD improvement at 2.5% with 2% standard deviation. For the two-sided test, we required a minimum 10-patient sample size in each group for detecting statistically significant differences in %FMD with power of 90% and α-type error of 5%. We expressed values as mean ± standard deviation for continuous variables and as numbers and percentages for categorical variables. We assessed differences between the two groups using Student's unpaired *t* or Mann–Whitney *U* tests for continuous variables and Chi-square or Fisher’s exact tests for categorical variables. We examined differences among multiple groups using the one-way analysis of variance followed by the Tukey–Kramer Honest significant difference test. We determined correlations between two variables using simple linear regression analyzes. A two-sided *P* value > 0.05 was considered statistically significant. We performed all statistical analyzes with SPSS version 14.0 (SPSS, Chicago, IL, USA).

## Results

### Control and MetS groups' baseline characteristics

Table 1 shows the patient baseline characteristics for the control (*n* = 68) and the MetS (*n* = 27) groups. Body weight (BW), body mass index (BMI), and abdominal circumference values in the MetS group were significantly higher than those in the control group. Overall, 89% of the patients in the MetS and 65% in the control groups had hypertension. The HbA1c and FPG levels did not differ significantly in the MetS and control groups. The mean HOMA-IR in the MetS group was significantly higher than that in the control group. Even though all patients were on statins, LDL-C and RLP-C levels in the MetS group were significantly higher than those in the control group.

**Table 1:** Baseline characteristics in the control and MetS groups

Variables	Control group (*n* = 68)	MetS group (*n* = 27)	*P* value
Age, years	67.5 ± 9.4	66.2 ± 9.8	0.745
BW (kg)	61.8 ± 6.9	75.9 ± 12.2	< 0.001
BMI (kg/m^2^)	22.9 ± 1.8	28.0 ± 3.2	< 0.001
Abdominal circumference (cm)	79.6 ± 5.1	97.1 ± 9.7	< 0.001
Current or past smokers, *n* (%)	45 (66)	19 (70)	0.694
Hypertension, *n* (%)	44 (65)	24 (89)	0.018
SBP (mmHg)	129.2 ± 15.4	137.3 ± 14.4	0.033
DBP (mmHg)	72.8 ± 11.2	83.9 ± 11.8	0.026
Glucose markers			
HbA1c (%)	5.9 ± 0.3	6.0 ± 0.6	0.279
Fasting plasma glucose (mg/dL)	101.3 ± 18.5	96.2 ± 11.7	0.613
Fasting plasma insulin (µIU/mL)	7.1 ± 4.0	10.2 ± 5.8	0.013
HOMA-IR	1.79 ± 1.17	2.47 ± 1.42	0.036
Use of statin, *n* (%)	68 (100)	27 (100)	1.000
Lipid markers			
Triglyceride (mg/dL)	134.2 ± 56.3	181.5 ± 86.4	< 0.001
LDL cholesterol (mg/dL)	98.5 ± 29.3	126.5 ± 30.8	0.019
HDL cholesterol (mg/dL)	49.8 ± 12.9	47.2 ± 12.7	0.392
RLP cholesterol (mg/dL)	4.6 ± 1.9	6.2 ± 2.9	0.032
Apolipoprotein A-I (mg/dL)	131.5. ± 29.8	137.4 ± 30.4	0.193
Apolipoprotein B (mg/dL)	83.9 ± 20.6	87.7 ± 19.9	0.581

Values for continuous variables are shown as mean ± SD; categorical variables are represented by number (percentage, %). Differences between groups were determined by the unpaired Student's *t* test or the Chi-squared test, with a statistical significance level of *P* < 0.05

*BW* body weight, *BMI* body mass index, *DBP* diastolic blood pressure, *HbA1c* hemoglobin A1c, *HDL* high-density lipoprotein, *HOMA-IR* homeostasis model assessment of insulin resistance, *LDL* low-density lipoprotein, *MetS* metabolic syndrome, *RLP* remnant lipoprotein, *SBP* systolic blood pressure, *SD* standard deviation

## Postprandial lipid and glucose metabolism before and after ezetimibe treatment

**Fig. 1:**
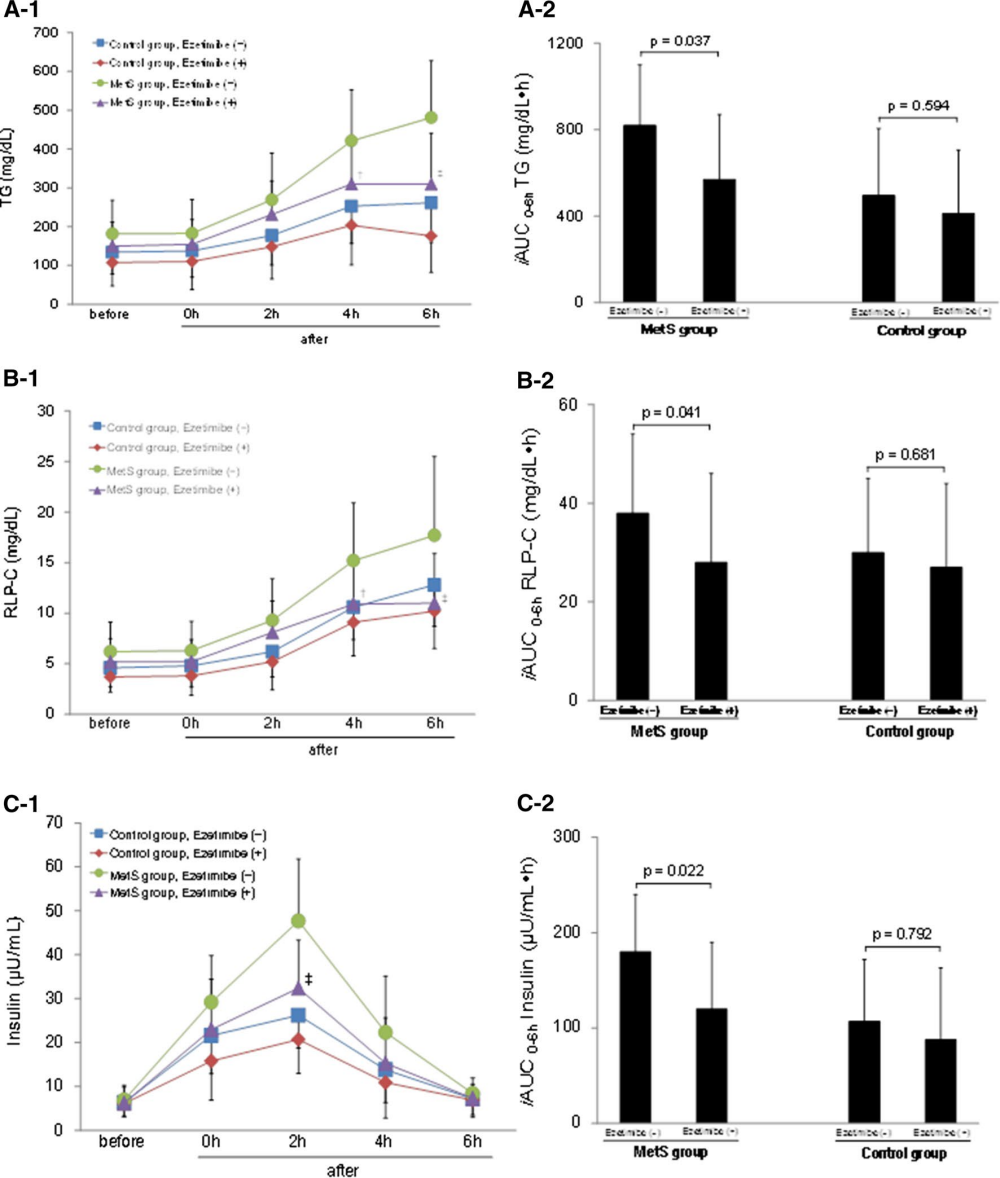
Postprandial changes in serum TG (**a-1**), RLP-C (**b-1**), and insulin (**c-1**) levels after the fat load ingestion; comparison of *i*AUCs for postprandial serum TG (**a-2**), RLP-C (**b-2**), and insulin (**c-2**) during the test. Data are expressed as mean ± SD. ^†^*P* < 0.05, ^‡^*P* < 0.01 compared with the same time-point values in the MetS group before ezetimibe treatment. *TG* triglyceride, *RLP-C* remnant-like particle cholesterol, *iAUC* incremental area under the curve, *MetS* metabolic syndrome

Table 2 summarizes changes in the lipid and glucose profiles during the oral fat loading test before and after ezetimibe treatment in the control and MetS groups. Serum TG and RLP-C levels changed significantly during the study. Other lipid markers did not differ meaningfully from the baseline. Plasma glucose and insulin levels also changed significantly during the loading test in both groups. Figure 1 (a1 and b1) shows the changes in serum TG and RLP-C levels during the oral fat loading test before and after ezetimibe treatment in the control and MetS groups. Pre-treatment serum TG and RLP-C levels continued to rise for 6 h after the high-fat meal in both groups. In the MetS group, pre-treatment serum TG and RLP-C levels were significantly higher than those in the control group 4 and 6 h after loading (4 h TG, *P* = 0.021; 6 h TG, *P* = 0.007; 4 h RLP-C, *P* = 0.025; 6 h RLP-C, *P* = 0.008). After 24 weeks of treatment, Ezetimibe significantly decreased the serum TG and RLP-C levels at 4 and 6 h after loading (4 h TG, *P* = 0.037; 6 h TG, *P* = 0.008; 4 h RLP-C, *P* = 0.031; 6 h RLP-C, *P* = 0.006). In the control group, we found no significant differences in serum TG and RLP-C levels at 4 and 6 h between values taken before and after ezetimibe treatment. The *i*AUC_0−6 h_ for serum TG and RLP-C decreased significantly after ezetimibe treatment in the MetS group (TG, from 820 ± 280 to 570 ± 300, *P* = 0.037; RLP-C, from 38 ± 16 to 28 ± 18, *P* = 0.041) (Fig. 1a2, b2). We found no significant differences in *i*AUC_0−6 h_ for either marker in the control group (TG, *P* = 0.594; RLP-C, *P* = 0.681) (Fig. 1a2, b2).

**Table 2:** Changes of lipid and glucose markers after the oral fat loading test

	Before	After			
		0 h	2 h	4 h	6 h
Triglyceride (mg/dL)					
Control group; ezetimibe (–)	134.2 ± 56.3	137.4 ± 67.9	177.2 ± 75.7	252.7 ± 95.8^‡^	261.5 ± 102.5^‡^
Control group; ezetimibe (+)	107.5 ± 60.3	110.2 ± 72.4	148.1 ± 82.7	203.5 ± 101.8^†^	175.9 ± 93.6
MetS group; ezetimibe (–)	181.5 ± 86.4	182.6 ± 87.0	269.1 ± 119.8^†^	421.0 ± 131.3^§^	481.0 ± 146.7^¶^
MetS group; ezetimibe (+)	149.6 ± 61.9	154.7 ± 63.3	231.1 ± 86.8^†^	310.0 ± 109.7^§^	309.7 ± 131.1^§^
RLP cholesterol (mg/dL)					
Control group; ezetimibe (–)	4.6 ± 1.9	4.8 ± 2.1	6.2 ± 2.5	11.6 ± 3.2^§^	13.8 ± 4.1^§^
Control group; ezetimibe (+)	3.7 ± 1.5	3.8 ± 1.9	5.2 ± 2.8	8.1 ± 3.3^§^	9.2 ± 3.7^§^
MetS group; ezetimibe (–)	6.2 ± 2.9	6.3 ± 2.9	9.3 ± 4.1	15.2 ± 5.7^¶^	17.7 ± 7.8^¶^
MetS group; ezetimibe (+)	5.2 ± 2.3	5.2 ± 2.2	8.1 ± 3.1	10.9 ± 4.0^§^	11.0 ± 4.9^§^
Glucose (mg/dL)					
Control group; ezetimibe (–)	101.3 ± 18.5	118.5 ± 23.6	121.9 ± 29.9^†^	98.9 ± 16.8	97.7 ± 11.4
Control group; ezetimibe (+)	101.7 ± 21.0	120.4 ± 20.9	122.7 ± 30.7^†^	96.7 ± 22.3	97.9 ± 13.6
MetS group; ezetimibe (–)	96.2 ± 11.7	128.8 ± 19.9^†^	132.2 ± 20.5^‡^	105.7 ± 23.8	97.9 ± 20.7
MetS group; ezetimibe (+)	93.7 ± 7.3	123.5 ± 20.3^‡^	129.7 ± 19.9^‡^	100.3 ± 21.5	98.5 ± 18.8
Insulin (μIU/mL)					
Control group; ezetimibe (–)	7.1 ± 4.0	21.6 ± 8.7^§^	26.2 ± 7.5^§^	13.8 ± 7.5^†^	7.2 ± 4.2
Control group; ezetimibe (+)	6.5 ± 2.9	15.8 ± 8.9^†^	20.7 ± 7.7^‡^	10.9 ± 8.1	6.9 ± 3.4
MetS group; ezetimibe (–)	10.2 ± 5.8	29.2 ± 10.6^‡^	47.7 ± 14.1^¶^	22.3 ± 12.8^†^	8.4 ± 3.5
MetS group; ezetimibe (+)	7.8 ± 3.3	23.0 ± 11.4^†^	35.4 ± 10.9^§^	15.3 ± 10.3^†^	7.3 ± 3.2

Values are shown as mean ± SD

*RLP* remnant lipoprotein, *SD* standard deviation

^†^*P* < 0.05, ^‡^*P* < 0.01, ^§^*P* < 0.005, ^¶^*P* < 0.001 compared with the value before the loading test in the same group

As shown in Fig. 1c1, the plasma insulin levels in both groups increased postprandially and reached peak levels at 2 h, returning to baseline levels by 4 or 6 h after the meal. Compared with the control group, the plasma insulin level in the MetS group at 2 h after the meal was significantly higher (*P* = 0.007). The *i*AUC_0−6 h_ for plasma insulin decreased significantly after ezetimibe treatment in the MetS group, but not in the control group (Fig. 1c2).

## Anthropometric parameters and insulin resistance markers before and after ezetimibe treatment

Table 3 summarizes the data on anthropometric parameters and insulin resistance markers in the control and MetS groups. BW, BMI, and abdominal circumference did not differ significantly before and after ezetimibe treatment in both groups.

**Table 3:** Anthropometric parameters and insulin resistance markers

	Control group (*n* = 68)			MetS group (*n* = 27)		
	Ezetimibe (–)	Ezetimibe (+)	*P* value	Ezetimibe (–)	Ezetimibe (+)	*P* value
BW (kg)	61.8 ± 6.9	60.3 ± 6.1	0.238	75.9 ± 12.2	73.8 ± 9.3	0.482
BMI (kg/m^2^)	22.9 ± 1.8	22.1 ± 1.6	0.128	28.0 ± 3.2	27.4 ± 2.3	0.331
Abdominal circumference (cm)	79.6 ± 5.1	79.3 ± 4.5	0.342	97.1 ± 9.7	94.3 ± 6.2	0.166
HOMA-IR	1.79 ± 1.17	1.64 ± 0.87	0.669	2.47 ± 1.42	1.83 ± 0.81	0.037
Fasting insulin (µIU/mL)	7.1 ± 4.0	6.5 ± 2.9	0.574	10.2 ± 5.8	7.8 ± 3.3	0.169
2-h insulin (µIU/mL)	26.2 ± 7.5	20.7 ± 7.7	0.183	47.7 ± 14.1	35.4 ± 10.9	0.008
*i*AUC_0−6 h_ insulin (µIU/mL h)	107 ± 65	88 ± 75	0.792	181 ± 62	121 ± 62	0.022

Values are shown as mean ± SD

*BW* body weight, *BMI* body mass index, *HOMA-IR* homeostasis model assessment of insulin resistance, *iAUC* incremental area under the curve

The mean HOMA-IR decreased significantly after ezetimibe treatment in the MetS group, but not in the control group. The mean baseline plasma insulin levels did not differ significantly before and after treatment in either group; however, the mean levels at 2 h after the load ingestion were significantly lower than those before treatment in the MetS group. The *i*AUC_0−6 h_ in plasma insulin decreased significantly after ezetimibe treatment in the MetS group, but not in the control group.

## Endothelial function before and after ezetimibe treatment

**Fig. 2:**
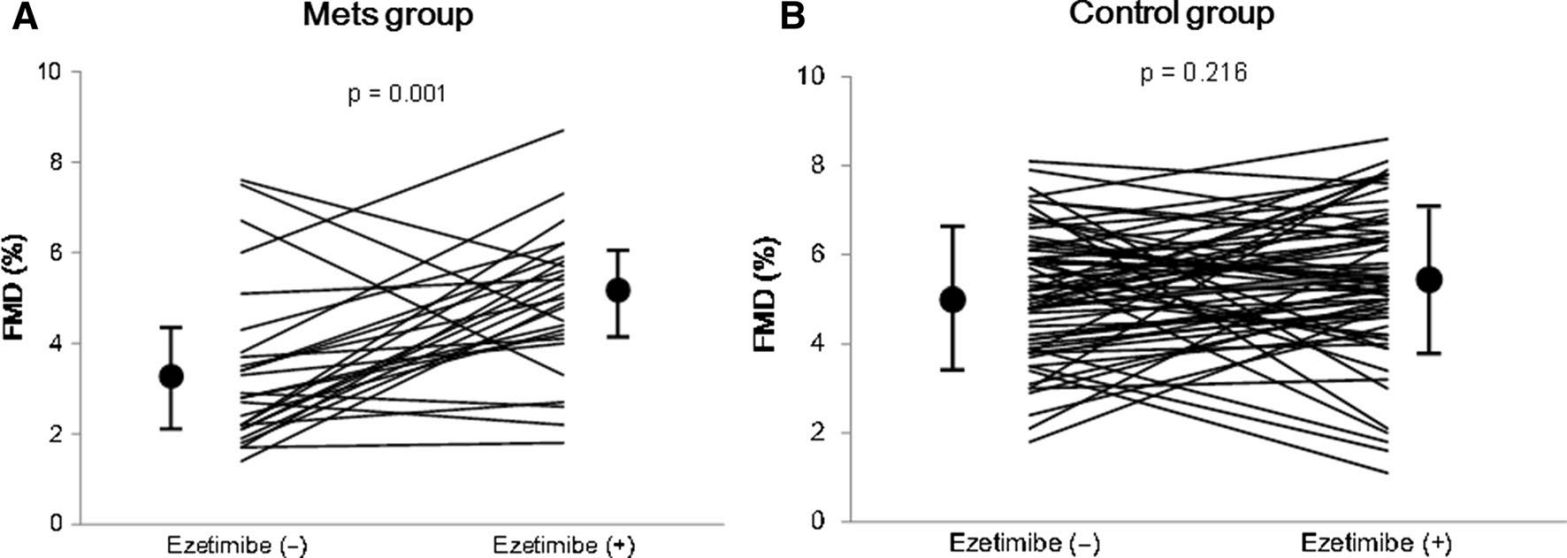
Changes in FMD measurements before and after ezetimibe treatment in the MetS (**a**) and control groups (**b**). *FMD* flow-mediated vasodilation, *MetS* metabolic syndrome

Figure 2a, b shows FMD changes measured before and after ezetimibe treatment in the MetS and control groups, respectively. The mean FMD increased significantly after ezetimibe treatment in the MetS group (from 3.4 ± 1.8% to 4.9 ± 1.6%, *P* = 0.002), but not in the control group (from 5.1 ± 1.4% to 5.4 ± 1.6%, *P* = 0.216).

## Associations between insulin resistance and postprandial TG or FMD changes in the MetS group

**Fig. 3:**
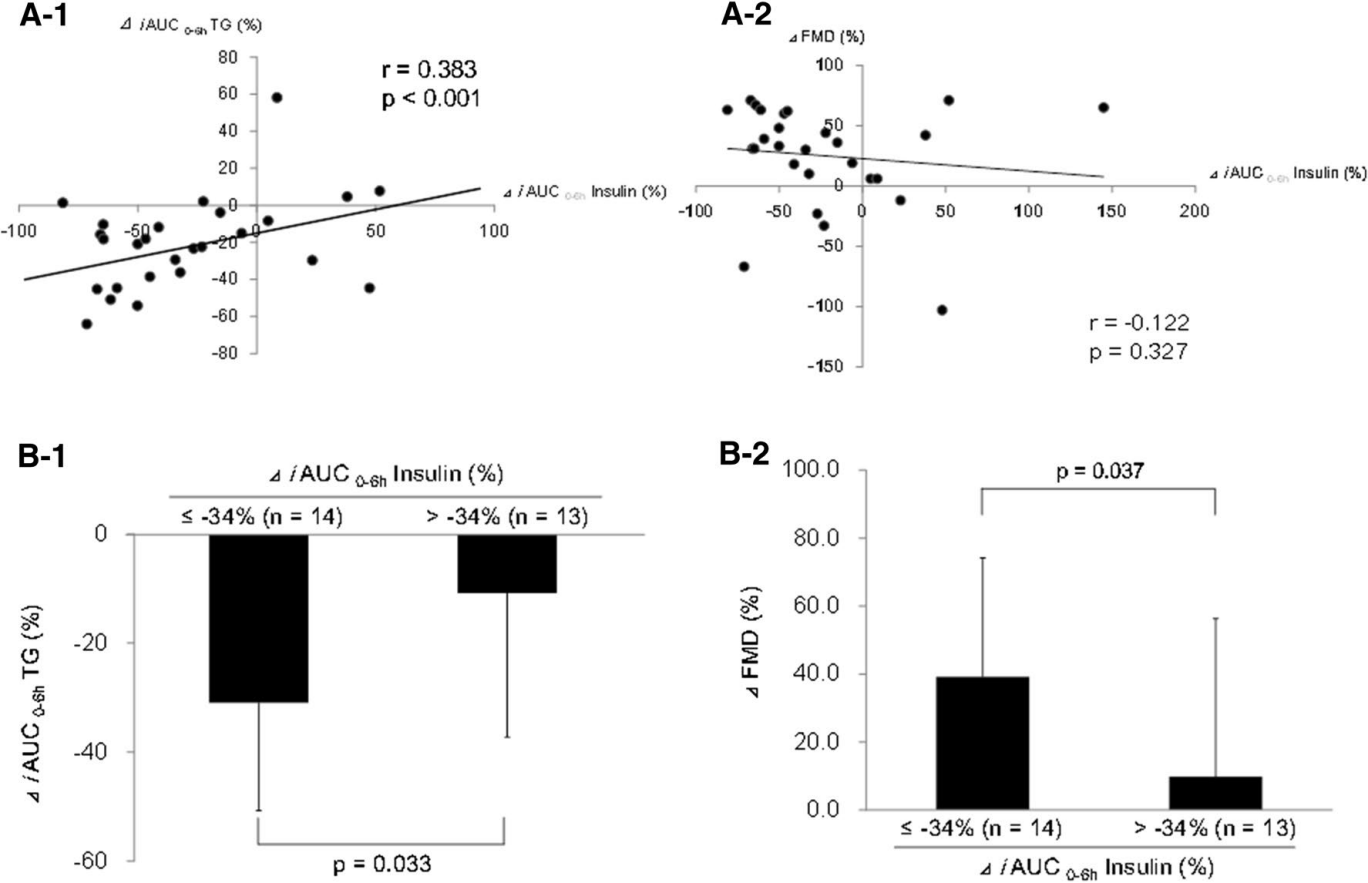
Correlation between the change ratio of ∆*i*AUC_0−6 h_ insulin and ∆*i*AUC_0−6 h_ TG (**a-1**) or ∆FMD (**a-2**) in the MetS group; comparison of reduction in ∆*i*AUC_0–6 h_ TG (**b-1**) and increase in ∆ FMD (**b-2**) between the MetS subgroups (below and above the median ∆*i*AUC_0−6 h_ insulin). *iAUC* incremental area under the curve, *TG* triglyceride, *FMD* flow-mediated vasodilation, *MetS* metabolic syndrome

Figure 3 shows the association between decreased insulin resistance and improved postprandial lipid markers or endothelial functions after ezetimibe treatment in the MetS group. The change ratio of *i*AUC_0−6 h_ insulin before and after the treatment (%∆ *i*AUC_0−6 h_ insulin) was correlated with that of *i*AUC_0−6 h_ TG (%∆ *i*AUC_0−6 h_ TG) (*r* = 0.383, *P* < 0.001) (Fig. 3a1), but not with the change ratio of FMD (%∆ FMD) (*r* = −0.122, *P* = 0.327) (Fig. 3a2).

When patients with MetS with %∆ *i*AUC_0−6 h_ insulin levels below and above the median value (– 34%) were compared, the %∆ *i*AUC_0–6 h_ TG was higher in the former group (− 31.0 ± 19.8% vs. − 10.8 ± 26.4%, *P* = 0.033) (Fig. 3b1). In addition, %∆ FMD was significantly higher in the below-median group than in the above-median group (39.2 ± 35.0 vs. 9.8 ± 46.5%, *P* = 0.037) (Fig. 3b2).

## Discussion

We identified three major findings in this study. First, the magnitude of postprandial serum TG or plasma insulin levels estimated by the *i*AUC_0−6 h_ after the fat load intake was higher in the MetS than in the control group, and the ezetimibe treatment reduced the postprandial hyperlipidemia and the hyperinsulinemia in the MetS group, but not in the control group. Second, FMD responses showed significant improvement after ezetimibe treatment in the MetS group, but not in the control group. Third, when comparing patients with MetS with higher or lower postprandial hyperinsulinemia reductions, those with higher reductions showed also greater postprandial hyperlipidemia and FMD reductions than those in the group with lower postprandial hyperinsulinemia reductions. These findings suggest that the ezetimibe effects on the postprandial lipid profile may be associated with decreased insulin resistance in patients with MetS.

Our results are compatible with studies showing reduced postprandial hyperlipidemia after ezetimibe treatment in patients with MetS [9, 30]. For example, Hajer et al. reported that treatment involving a combination of simvastatin/ezetimibe (10 mg/10 mg, once daily) for 6 weeks improved postprandial TG levels integrated as AUCs after ingestion of a test meal containing standardized fats (50 g/m^2^) in men with obesity and MetS [30]. Moreover, Hiramitsu et al. reported that ezetimibe treatment for 4 weeks significantly reduced postprandial hyperlipidemia after ingestion of a high-fat and high-glucose meal (lipid 61.4 g; carbohydrate 79.8 g) in patients with obesity or hypertriglyceridemia [9]. We also found that the postprandial plasma insulin responses improved 24 weeks after ezetimibe treatment in patients with MetS; these results were consistent with others showing ezetimibe reduced postprandial hyperinsulinemia [9].

Ezetimibe has been shown to improve insulin resistance in small-animal models and humans [19, 20, 30–32]. Ezetimibe upregulates small heterodimer partner (SHP) expression in the liver (SHP silencing worsens insulin resistance) protecting the liver against the SHP downregulation that occurs in mice after a high-fat diet [30]. In addition, ezetimibe improved the insulin response after intraperitoneal glucose injection in a Zucker obese rat model and enhanced insulin signaling in cultured hepatocytes [19]. In humans, studies have shown that ezetimibe also ameliorates liver pathology and insulin resistance in patients with nonalcoholic fatty liver disease [31]. Moreover, ezetimibe improves not only lipid profiles, but also atherogenic factors and biomarkers, such as hepatocyte growth factor and insulin resistance, in patients with obesity and hypercholesterolemia [20]. Twenty-four weeks of ezetimibe treatment, combined with standard diet and exercise therapy, reduced body weight and improved atherogenic lipid profiles, but also reduced HOMA-IR in patients with MetS not-taking lipid-lowering drugs [32]. Our data are consistent with those last two studies [20, 32], suggesting that ezetimibe may play a unique pathophysiological role in the treatment of MetS. Of particular interest, we observed that the percent reductions in postprandial hyperlipidemia and hyperinsulinemia were correlated in our non-diabetic patients with MetS. Although we cannot confirm whether this association is general or specific to ezetimibe treatment, this is the first report showing an association between lipid metabolism and insulin resistance in a postprandial state before and after ezetimibe therapy.

Ezetimibe has effects on the lipid metabolism in the small intestine other than its cholesterol absorption inhibition via NPC1L1. Sandoval et al. investigated the molecular mechanisms of ezetimibe on postprandial hyperlipidemia in MetS using CD36 knockout mice and established a model for evaluating postprandial hypertriglyceridemia in a MetS environment [33]. They demonstrated that ezetimibe reduces the expression of fatty acid transport protein (FATP)4 associated with the absorption of long-chain fatty acids through enterocytes and decreases the gene expression of Apo B48 (the core structural protein for chylomicron particles) [33]. Thus, even though the mechanisms explaining how ezetimibe reduces postprandial hyperlipidemia in humans are not completely clear, the molecular targets for ezetimibe seem to be important not only for cholesterol absorption, but also for fatty acids and apolipoprotein synthesis regulation for TG production and subsequent chylomicron formation, at least in small animals. Insulin resistance is relevant to the regulation of molecules like FATP4 or Apo B48, which are involved in pathophysiological cholesterol and fatty acid incorporation into chylomicrons in the small intestine [34, 35]. Therefore, the effects of ezetimibe on postprandial lipid dysmetabolism may be partially explained by mechanisms, other than NPC1L1 inhibition, associated with decreased insulin resistance.

We previously demonstrated that postprandial hyperlipidemia is associated with insulin resistance in patients with DM and CAD without MetS [21]. In here, we investigated the effects of ezetimibe on postprandial lipid dysmetabolism and insulin resistance in non-diabetic patients with CAD and MetS. Our findings support the evidence that insulin resistance, manifesting as fasting or postprandial hyperinsulinemia, is the driving force behind lipid dyslipidemia in patients with MetS before overt DM development [36]. Therefore, the early combination of ezetimibe with a statin may not only help reduce LDL-C levels, but may also decrease insulin resistance, improving the prognosis for patients with MetS. A large randomized controlled trial, the improved reduction of outcomes: Vytorin Efficacy International Trial, demonstrated the efficacy of the ezetimibe–statin combination therapy for reducing the occurrence of major adverse cardiovascular events (MACEs) in patients with DM who had experienced myocardial infarction [37]. Moreover, Katsiki et al. suggested that the ezetimibe MACE benefits are more prominent in patients with MetS and DM than in those without DM [38]. These anti-atherosclerotic effects, which seem to reduce postprandial hyperlipidemia, may be partially explained by a reduction in insulin resistance in high-risk patients (such as those with DM or MetS).

## Study limitations

We are aware of our study's limitations. First, we conducted it at a single facility with a relatively low number of patients, and statistical biases may have been introduced, although our results were statistically significant. Second, we did not use a 75 g oral glucose tolerance test to ensure exclusion of patients with DM and relied only on ADA criteria. Third, the improvement of FMD value after ezetimibe treatment in MetS group was statistically significant, but lower than the expected value. The possibility exists that the study may have been underpowered to detect clinically meaningful differences before and after ezetimibe treatment in MetS group.

## Conclusions

This study demonstrated that ezetimibe significantly improved endothelial function and reduced both postprandial TG and insulin levels in patients with MetS. The potential association between decreased insulin resistance and reduced postprandial lipid dysmetabolism suggests that ezetimibe is the possible drug with regard to the vascular protective effects in such patients due to reduction in both insulin level and postprandial hyperlipidemia.
